# Biosynthesized Calcium Peroxide Nanoparticles as a Multifunctional Platform for Liver Cancer Therapy

**DOI:** 10.3390/ijms26104696

**Published:** 2025-05-14

**Authors:** Sen Wu, Siqi Li, Xin Xia, Gen Zhang, Ting Wang

**Affiliations:** 1 Department of Cell Biology, School of Basic Medicine, Nanjing Medical University, Nanjing 211166, China; senwu@stu.njmu.edu.cn (S.W.); lsq001022@163.com (S.L.); 2 Department of Human Anatomy, School of Basic Medicine, Nanjing Medical University, Nanjing 211166, China; xiaxin@njmu.edu.cn

**Keywords:** biosynthetic methods, CaO_2_ NPs, biocompatibility, prolonged retention, drug loading

## Abstract

To overcome the limitations associated with chemically synthesized nanoparticles in cancer therapy, researchers have increasingly focused on developing nanoparticles with superior biocompatibility and prolonged tumor retention using biosynthetic methods. In this study, we first identified the presence of calcium peroxide nanoparticles (CaO_2_ NPs) in the blood of individuals who had ingested calcium gluconate. Furthermore, the dropwise addition of calcium gluconate to human serum resulted in the spontaneous self-assembly of CaO_2_ NPs. Next, following tail vein injection of fluorescently labeled CaO_2_ NPs into subcutaneous tumor-bearing nude mice, we observed that the nanoparticles exhibited prolonged accumulation at the tumor sites compared to other organs through visible-light imaging. Immunofluorescence staining demonstrated that CaO_2_ NPs co-localized with vesicular transport-associated proteins, such as PV-1 and Caveolin-1, as well as the albumin-binding-associated protein SPARC, suggesting that their transport from tumor blood vessels to the tumor site is mediated by Caveolin-1- and SPARC-dependent active transport pathways. Additionally, the analysis of various organs in normal mice injected with CaO_2_ NPs at concentrations significantly higher than the experimental dose showed no apparent organ damage. Hemolysis assays indicated that hemolysis occurred only at calcium concentrations of 300 µg/mL, whereas the experimental concentration remained well below this threshold with no detectable hemolytic activity. In a subcutaneous tumor-bearing nude mouse model, treatment with docetaxel-loaded CaO_2_ NPs showed a 68.5% reduction in tumor volume compared to free docetaxel (DTX) alone. These novel biosynthetic CaO_2_ NPs demonstrated excellent biocompatibility, prolonged retention at the tumor site, safety, and drug-loading capability.

## 1. Introduction

Cancer remains a major global health burden, characterized by uncontrolled cell proliferation, invasion, and metastasis, leading to significant morbidity and mortality. Liver cancer, in particular, poses a critical health challenge, with approximately 900,000 new cases and 830,000 deaths reported worldwide in 2020 [1]. Although conventional treatments such as surgery, radiotherapy, and chemotherapy have made significant progress, their efficacy remains limited due to a lack of specificity and severe side effects.

To improve chemotherapy outcomes, an increasing number of studies are focusing on nanocarriers for drug delivery. In particular, metal-based nanoparticles achieve improved synergistic effects by utilizing metal-specific cytotoxicity [2], immune activation [3], and other mechanisms, thereby enhancing therapeutic outcomes.

However, the majority of current medical nanoparticles are synthesized chemically, a process that often involves toxic reagents and complex manufacturing steps [4]. These chemically synthesized nanoparticles face challenges in clinical translation due to poor biocompatibility.

Over the past two decades, researchers have identified that bacteria, viruses, fungi, and other lower organisms can effectively utilize metal ions to synthesize nanoparticles [5,6]. Studies have shown that when mice are fed a zinc acetate solution, ZnO nanocrystals, referred to as bio-ZnO NCs, are detected in their blood [7]. With the rise of nanotechnology, this phenomenon has been redefined as a biosynthesis method for nanoparticles. In this process, organisms use biomolecules such as proteins, enzymes, and polysaccharides for reduction, stabilization, or templating, converting metal ions into nanoparticles with specific sizes, shapes, and functions [6], this process typically occurs under mild conditions, with key mechanisms involving redox reactions, nucleation and growth, and stabilization. Nanoparticles derived from biological systems exhibit superior biocompatibility and safety, making them more promising for future applications compared to chemically synthesized nanoparticles. Recent studies have increasingly reported the isolation and purification of such nanoparticles from biological sources. In our previous research, we identified the presence of platinum nanoparticles in the blood of patients undergoing cisplatin chemotherapy and successfully synthesized them in vitro using a biosynthetic approach [8]. Furthermore, we explored the potential for the in vivo formation of nanoparticles from other metal elements.

Calcium, as an essential metal element in the human body, plays a critical role in tumor biology [9]. Studies have demonstrated that calcium-based nanoagents can enhance CD8^+^ T cell infiltration in tumor tissues [10], inhibit tumor invasion [11], and induce tumor cell death by triggering calcium overload [12,13,14].

Given the promotive role of calcium ions in liver cancer treatment [15,16], a recent study has demonstrated that CaO_2_ NPs can alleviate the hypoxic tumor microenvironment by generating oxygen in situ at tumor sites, thereby enhancing the efficacy of photodynamic therapy (PDT) in breast cancer [17]. This finding further underscores the therapeutic potential of CaO_2_ NPs in cancer treatment. We are committed to addressing the challenges of tumor targeting and biocompatibility associated with CaO_2_ NPs. To achieve this, we focused on the presence of calcium-based nanoparticles in biological systems. Notably, we discovered CaO_2_ NPs in individuals who had ingested calcium gluconate and subsequently synthesized them in vitro using a similar biosynthetic method. This type of biosynthesized calcium-based nanoparticles has not been reported yet. They exhibit the inhibitory effect of calcium ions on liver cancer cells, as well as the tumor-targeting ability and drug delivery function of the nanocarriers, highlighting their strong potential for cancer therapy.

In this study, we characterized their size and composition, and investigated their retention ability at tumor sites, synergistic therapeutic effects in drug delivery, biocompatibility, and safety. This research aims to develop a novel nanoparticle-based drug delivery platform for enhanced liver cancer treatment.

## 2. Results

### 2.1. Biosynthesis and Characterization of CaO_2_ NPs

**Scheme 1:**
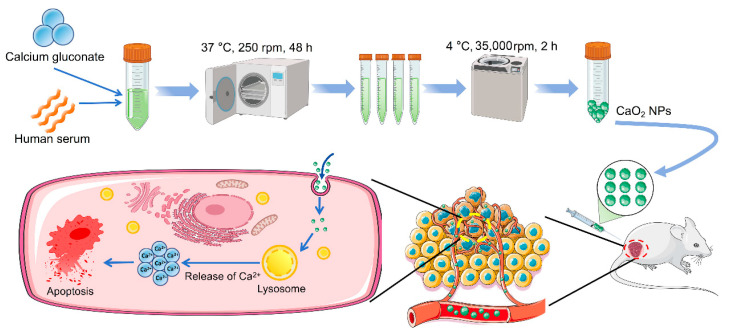
The biosynthesis of CaO_2_ NP nanocarriers for the treatment of liver cancer.

**Figure 1:**
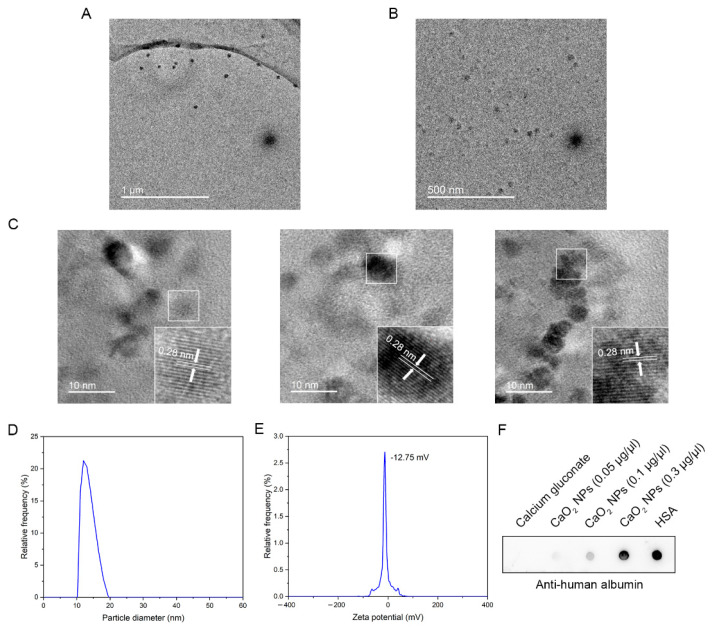
Characterization analysis of CaO_2_ NPs. (**A**) Representative TEM image of CaO_2_ NPs isolated from human blood, scale bar = 1 μm. (**B**) Representative TEM image of CaO_2_ NPs biosynthesized by calcium gluconate and human serum in vitro, scale bar = 500 nm. (**C**) High-resolution transmission electron microscopy of CaO_2_ NPs (lattice: 0.28 nm), scale bar = 10 nm. (**D**) Size distribution of CaO_2_ NPs in PBS as characterized by DLS. (**E**) Negative CaO_2_ NP surface charge at −12.75 mV in phosphate buffer. (**F**) Dot blot assay to detect albumin in isolated CaO_2_ NPs. Human serum albumin (HSA) served as positive control, calcium gluconate used as negative control.

Nanoparticles were extracted from the serum of volunteers who had taken calcium gluconate through ultracentrifugation at 35,000 rpm. Transmission electron microscopy (TEM) images revealed that the particles were uniformly distributed, with no aggregation, and exhibited a roughly spherical shape (Figure 1A). Due to the need for a larger quantity of nanoparticles and the simulation of the in vivo environment in experiments, we synthesized the nanoparticles in vitro using calcium gluconate and human serum following the method outlined in Scheme 1. Through transmission electron microscopy, we observed the presence of identical nanoparticles (Figure 1B). The lattice fringe on the surface of the nanoparticles obtained by both methods were 0.28 nm apart (Figure 1C), which closely matched the lattice spacing of calcium peroxide [18]; the result in Figure 1D shows that the average particle size of the particles was 12 nm, consistent with the size observed under TEM, indicating well-dispersed particles without clustering. The Zeta potential was −12.75 mV, confirming the good stability of the nanoparticles (Figure 1E). Given that the particles carry a negative surface charge, we hypothesize that their surfaces are coated with proteins. Based on our previous studies, where albumin was identified as the predominant component of the protein corona formed around platinum and zinc oxide nanoparticles [8,19], and considering that albumin is the most abundant protein in human serum, we employed a dot blot assay to confirm that albumin was also present on the surface of the CaO_2_ NPs (Figure 1F).

### 2.2. CaO_2_ NPs Exhibit Prolonged Retention at the Tumor Site

**Figure 2:**
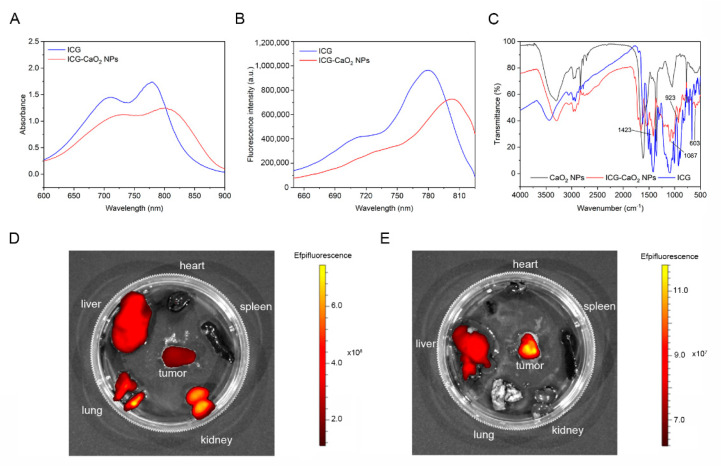
Tumor targeting of CaO_2_ NPs. (**A**) Absorbance spectra of ICG and ICG-CaO_2_ NPs. (**B**) Excitation spectra of ICG and ICG-CaO_2_ NPs. (**C**) FTIR spectra of ICG, CaO_2_ NPs, and ICG-CaO_2_ NPs. (**D**) Representative in vitro fluorescence images of tumors and major organs at 1 h after intravenous treatment of tumor-bearing mice with ICG-CaO_2_ NPs (10 mg/kg calcium, *n* = 3 mice). (**E**) Representative in vitro fluorescence images of tumors and major organs at 72 h after intravenous treatment of tumor-bearing mice with ICG-CaO_2_ NPs (10 mg/kg calcium, *n* = 3 mice).

We conjugated ICG to the surface of the CaO_2_ NPs via an EDC/NHS-mediated condensation reaction. UV–visible spectroscopy revealed characteristic absorption peaks of the ICG-CaO_2_ NPs at 730 nm and 800 nm, which are consistent with the absorption wavelengths of free ICG (Figure 2A). Fluorescence spectroscopy showed that the excitation wavelength of the ICG-CaO_2_ NPs was approximately 800 nm, similar to that of free ICG (Figure 2B). Fourier transform infrared (FTIR) spectroscopy was used to analyze the interaction between the ICG and CaO_2_ NPs and assess the stability of the ICG after loading onto the nanoparticles. The FTIR spectrum of the ICG-CaO_2_ NPs retained the characteristic peaks of ICG, suggesting that the ICG remained stable after encapsulation. Notably, the ICG-CaO_2_ NPs exhibited peaks at 603 cm^−1^, 923 cm^−1^, 1087 cm^−1^, and 1423 cm^−1^, which corresponded to those of the ICG spectrum (Figure 2C), confirming the successful loading of ICG onto the CaO_2_ NPs. These results demonstrate the synthesis of ICG-CaO_2_ NPs with near-infrared fluorescence properties.

To evaluate the retention capability of the CaO_2_ NPs at the tumor site, we used in vivo small animal imaging to track their biodistribution in mice. Based on the recommended daily calcium intake of 800 mg for adults [20], we estimated the required dosage of CaO_2_ NPs, with a concentration of 10 mg of calcium per kg of body weight for mice, or 150 µg of calcium per milliliter of blood. ICG-CaO_2_ NPs were injected intravenously into nude mice bearing subcutaneous MHCC97-H cell tumors. An hour post-injection, fluorescence signals were observed in the liver, tumor, lungs, and kidneys of the tumor-bearing nude mice (Figure 2D); after 72 h, the fluorescence signals were predominantly concentrated in the tumors and livers of the tumor-bearing nude mice, with significantly higher intensity in the tumor compared to the liver (Figure 2E). These results indicate that the CaO_2_ NPs exhibit prolonged retention at the liver cancer site.

### 2.3. CaO_2_ NPs Enter Tumor Tissues Through Active Pathways

The above results have demonstrated that CaO_2_ NPs can effectively accumulate in liver cancer tissues. We further investigated how CaO_2_ NPs traverse tumor vasculature to enter tumor tissues. Previous studies suggest that albumin-coated metal nanoparticles cross blood vessels via active transport and enter cells through endocytosis [19,21,22]. To explore this mechanism, we selected PV-1, a tumor endothelial cell-specific marker, along with Caveolin-1, which is known to facilitate albumin endocytosis by endothelial cells [23], and SPARC, a protein that specifically binds to albumin.

**Figure 3:**
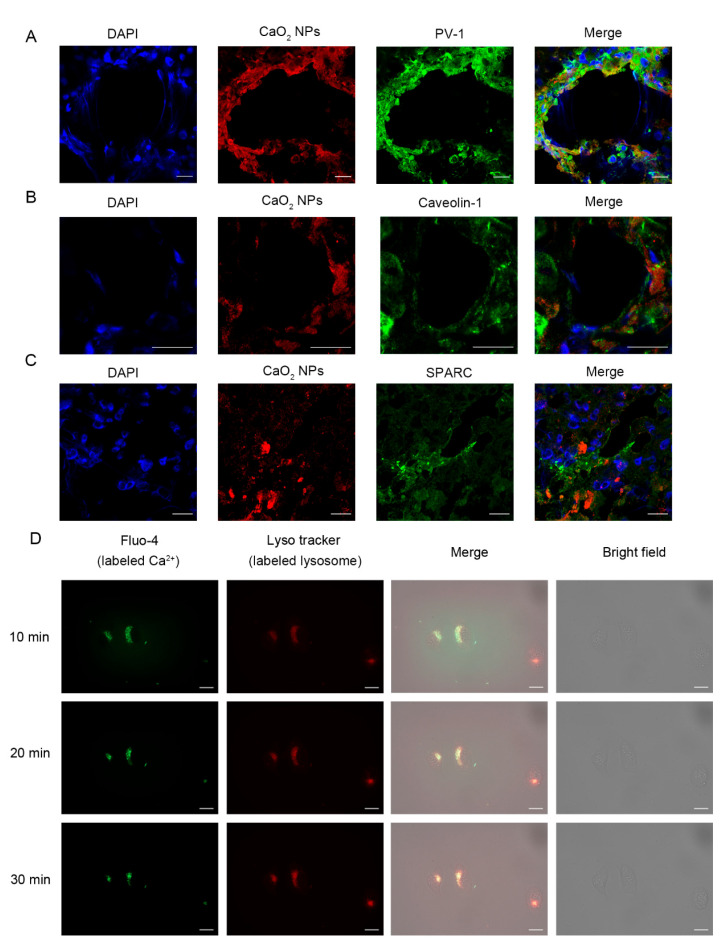
CaO_2_ NPs enter tumor tissues through active pathways. Representative immunofluorescence images of tumor sections from the tumor-bearing mice treated with CaO_2_ NPs (10 mg/kg calcium). (**A**) Tumor sections stained with anti-PV-1 antibodies (green), CaO_2_ NPs (red), and DAPI (blue), and the merged image (yellow). Scale bar = 20 μm. (**B**) Tumor sections were stained with anti-Caveolin-1 antibodies (green), CaO_2_ NPs (red), and DAPI (blue). Scale bar = 20 μm. (**C**) Tumor sections stained with anti-SPARC antibodies (green), CaO_2_ NPs (red), and DAPI (blue), and the merged image (yellow). Scale bar = 20 μm. (**D**) Live-cell imaging showing lysosomes (red) and calcium ions (green) in MHCC 97-H cells incubated with 150 mg/L CaO_2_ NPs; scale bar = 10 μm.

We cryosectioned the tumors and stained them with anti-PV-1 antibody, a marker of vesicles, revealing that the CaO_2_ NPs remained co-localized with vascular endothelial cells until their entry into the stroma (Figure 3A), suggesting that CaO_2_ NPs penetrate via a trans-endothelial pathway. Furthermore, the co-localization of CaO_2_ NPs with Caveolin-1 and SPARC was observed in tumor sections (Figure 3B,C). This highlights that the extravasation of CaO_2_ NPs is primarily driven by active transport mechanisms.

We observed the process of CaO_2_ NPs entering cells using a live-cell imaging system and used LysoTracker Red to label acidic lysosomal structures, while Fluo-4 was employed to monitor intracellular Ca^2+^ levels. As shown in Figure 3D, after adding CaO_2_ NPs for 10 min, we observed a progressively stronger green fluorescence that gradually filled the entire lysosome. This indicates that exogenous CaO_2_ NPs are internalized into lysosomes for digestion. Based on these observations, we hypothesize that the endocytosis of CaO_2_ NPs by cancer cells leads to lysosomal swelling, which progressively disrupts lysosomal membrane integrity. Ultimately, this leads to lysosomal dysfunction, resulting in cell apoptosis, as presented below.

### 2.4. The Synergistic Cytotoxic Effect of CaO_2_ NPs as Nanocarriers in Liver Cancer Cells

**Figure 4:**
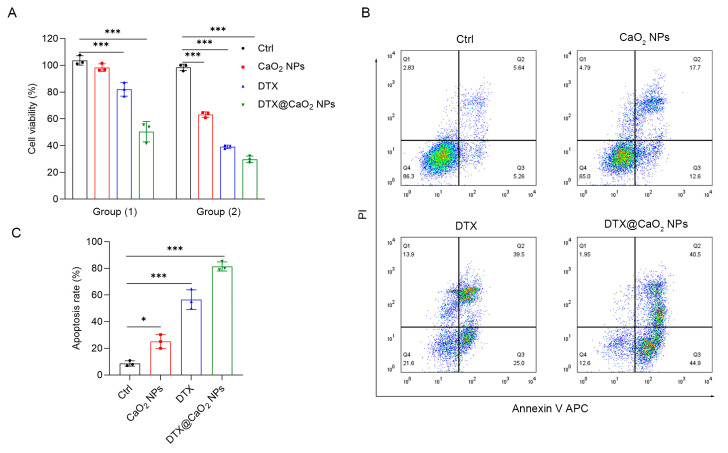
CaO_2_ NPs as nanocarriers promote apoptosis in liver cancer cells. (**A**) The Cell Counting Kit (CCK-8) assay of MHCC 97-H cells. Group (1): the cells were treated with CaO_2_ NPs (150 mg/L), DTX (37.5 mg/L), or DTX@CaO_2_ NPs (containing 37.5 mg/L DTX) and tested after incubation for 48 h. Group (2): The cells were treated with CaO_2_ NPs (300 mg/L), DTX (75 mg/L), or DTX@CaO_2_ NPs (containing 75 mg/L DTX) and tested after incubation for 48 h. The bars represent the results from three independent experiments (*n* = 3); *** *p* < 0.001. (**B**) The flow cytometry analysis using Annexin V/PI staining. MHCC 97-H cells were treated with 150 mg/L CaO_2_ NPs, 37.5 mg/L DTX, or DTX@CaO_2_ NPs (containing 37.5 mg/L DTX) for 24 h. (**C**) The quantitative analysis of the apoptotic cells in B (*n* = 3); * *p* < 0.033, *** *p* < 0.001.

We next loaded the chemotherapeutic drug docetaxel into the nanoparticles to evaluate its cytotoxic effects on liver cancer cells. The CCK-8 assay demonstrated that, at low concentrations, the drug-loaded nanoparticle group exhibited the strongest inhibition of tumor cell viability, with a reduction of 49.6%, compared to the other groups. At high concentrations, the drug-loaded nanoparticle group achieved a cell viability inhibition rate of 70% (Figure 4A). The flow cytometry results showed that the apoptosis rates induced by the CaO_2_ NPs and DTX in liver cancer cells were 12.6% and 25%, respectively, while the DTX@CaO_2_ NPs induced the highest apoptosis rate in liver cancer cells, reaching 44.9% (Figure 4B,C). Taken together, these findings suggest that CaO_2_ NPs and DTX exert a synergistic effect, with DTX@CaO_2_ NPs promoting tumor cell apoptosis more effectively than DTX alone.

### 2.5. The Synergistic Therapeutic Effect of CaO_2_ NPs as Nanocarriers In Vivo

**Figure 5:**
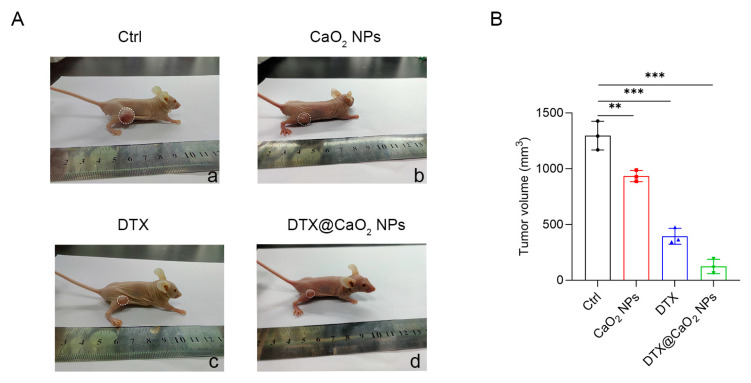
CaO_2_ NPs as drug delivery nanocarriers for liver tumor treatment. (**A**) Influence of different treatment methods on volume size of nude mouse transplanted tumor: (**a**) control group (inject normal saline); (**b**) inject CaO_2_ NPs (10 mg/kg calcium); (**c**) inject 3 mg/kg DTX; (**d**) inject DTX@CaO_2_ NPs (3 mg/kg DTX). (**B**) Tumor volume on day 28 after different treatments (*n* = 3 mice). ** *p* < 0.002, *** *p* < 0.001.

The antitumor efficacy of CaO_2_ NPs was evaluated in a subcutaneous tumor-bearing nude mouse model. Subcutaneous tumor-bearing nude mice were divided into four groups: Group I (Ctrl), Group II (CaO_2_ NPs), Group III (DTX), and Group IV (DTX@CaO_2_) NPs. All four groups received tail vein injections, and tumor volumes were measured using a caliper and photographed for confirmation (Figure 5A,B). Compared to the control group, tumor size in the CaO_2_ NPs group was reduced by 28%. Similarly, the DTX@CaO_2_ NPs group exhibited a 68.5% reduction in tumor size compared to the DTX-only group. Importantly, the CaO_2_ NPs enhanced the antitumor effect of DTX, with the smallest tumor volume observed in the DTX@CaO_2_ NPs group. These findings further underscore the enhanced therapeutic efficacy of DTX@CaO_2_ NPs against liver tumors compared to free DTX, highlighting the potential of CaO_2_ NPs as highly effective nanocarriers for anticancer drug delivery.

### 2.6. Biocompatibility and Safety of CaO_2_ NPs

**Figure 6:**
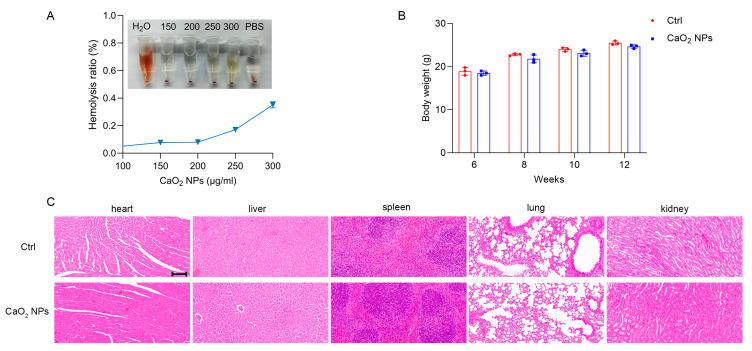
The biosafety assessment of CaO_2_ NPs. (**A**) The hemolysis rate of CaO_2_ NPs. PBS served as the positive control, while deionized water served as the negative control. (**B**) The body weights of the BALB/c mice treated with CaO_2_ NPs (blue) or saline vehicle control (red) for 6 weeks. The mice were treated with CaO_2_ NPs (10 mg/kg calcium) by intravenous injection once per week, and their weights were noted every two weeks. (**C**) The histological analysis of the BALB/c mice heart, liver, spleen, lungs, and kidneys (CaO_2_ NPs (20 mg/kg calcium) treatment by tail vein for 6 weeks). Representative images are shown (*n* = 3). Scale bar = 100 μm.

We evaluated the systemic toxicity of CaO_2_ NPs through hemolysis assays and histological analysis. The hemolysis assays indicated that CaO_2_ NPs (300 µg/mL calcium) caused negligible damage to mouse red blood cells (Figure 6A). Additionally, there were no noticeable differences in body weight changes between the treatment and control groups during the injection period (Figure 6B). To further investigate potential organ toxicity, key organs (heart, liver, spleen, lungs, and kidneys) were collected and subjected to H&E staining; histological analysis revealed no significant signs of toxicity or adverse effects in any of the major organs following CaO_2_ NP (20 mg/kg calcium) treatment (Figure 6C). Collectively, these findings suggest that CaO_2_ NPs exhibit favorable biocompatibility and safety in vivo.

## 3. Discussion

Zeng et al. identified the presence of platinum and zinc oxide nanoparticles in the human body [8,19]. Interestingly, not only heavy metals but also intrinsic metallic elements of the human body can form nanoparticles in vivo. Our research further demonstrates that calcium can also form biological nanoparticles within organisms, as evidenced by electron microscopy. Building on this, we developed tumor-specific, highly selective, and low-toxicity calcium peroxide nanoparticles (CaO_2_ NPs) through in vitro simulated biosynthesis. While chemically synthesized calcium oxide nanoparticles face similar clinical application challenges as other nanoparticles, ongoing studies on biosynthetic metal nanoparticles have begun to address these concerns.

Among the various factors influencing the retention and elimination of nanoparticles, size plays a crucial role [24,25]. The glomerular filtration barrier retains nanoparticles and proteins larger than 6–8 nm within the body, while smaller nanoparticles and proteins are rapidly cleared by the kidneys [26]. CaO_2_ NPs, with a size of 12 nm, exhibit prolonged retention at tumor sites, minimizing rapid elimination. Importantly, their relatively small size facilitates uniform diffusion throughout tumor tissue, enabling deeper penetration into distal tumor regions. In contrast, nanoparticles with larger sizes tend to accumulate at the tumor periphery, as they are unable to penetrate deeper into the tumor mass due to obstruction by the extracellular matrix fibers. Additionally, the surface charge of nanoparticles also influences their behavior in vivo [27]. Negatively charged CaO_2_ NPs reduce aggregation and exhibit efficient drug delivery profiles, advantageous for sustained therapeutic effects [28].

This study demonstrated that CaO_2_ NPs primarily induce cell death through apoptosis. However, the underlying mechanism requires further investigation and may be related to the intracellular effects of calcium ions. Previously chemically synthesized calcium-based nanomaterials disrupt calcium homeostasis, inducing calcium overload, which promotes tumor cell death and inhibits tumor growth [16,29,30,31]. Excessive intracellular calcium disrupts cell membrane integrity, activating phospholipases that degrade phospholipid components, leading to membrane rupture and necrosis [32,33]. Additionally, elevated calcium levels can induce excessive autophagy by activating autophagy-related proteins (ATGs), resulting in numerous autophagosomes [34,35,36]. The overconsumption of cellular materials and energy ultimately leads to cell death. Moreover, calcium ions act as signaling molecules that trigger antitumor immune responses, promoting M1 macrophage polarization and dendritic cell maturation within the tumor microenvironment [37,38]. Targeting key molecules in these pathways for small-molecule drug design will be a focus of our future research. Nonetheless, the interactions between calcium-based nanomaterials and biological systems, as well as their impact on cellular processes, require further investigation; this is critical for improving their clinical safety and efficacy.

The primary pathway for nanoparticle entry into cells is endocytosis. Following internalization, the nanoparticles are encapsulated within endocytic vesicles, which subsequently mature by fusing with early endosomes and then late endosomes. This intracellular trafficking process typically culminates in the delivery of nanoparticles to lysosomes [39], where they are exposed to an acidic environment and hydrolytic enzymes. In liver cancer cells, the overexpression of the lysosomal membrane protein TRPML1 enhances calcium ion release [40,41]. Understanding this pathway is critical, as it influences the fate of the nanoparticles, including their degradation, drug release behavior, and potential for endosomal escape.

Our results indicate that DTX@CaO_2_ NPs exhibit superior antitumor efficacy compared to free DTX, likely due to the delivery properties of CaO_2_ NPs. These properties include the enhanced uptake of DTX by tumor cells and the increased accumulation of the drug within tumor tissues. In addition, the accumulation of CaO_2_ NPs promotes apoptosis in liver cancer cells and exerts a synergistic anticancer effect when combined with DTX. As a biocompatible delivery system, CaO_2_ NPs hold potential for combination with other chemotherapy agents for tumor treatment. However, there are some limitations in this study. Firstly, the number of samples in the animal experiments is relatively small, which may reduce the statistical power of the results, especially in group comparisons. Furthermore, given the inherent physiological and pathological differences between animal models, the chosen model may not fully represent the clinical conditions in humans. Therefore, future studies should consider increasing the sample number and incorporating a more diverse range of animal models to enhance the clinical transformation potential of the findings. Moreover, we do not quantify the proportion of nanoparticles transported via active transport versus passive diffusion. Future research should address this limitation by employing cardiac perfusion fixation to immobilize tumors, facilitating the quantification of extravasated nanoparticles. Furthermore, employing transmission electron microscopy (TEM) and 3D microscopy to investigate the vascular ultrastructure and analyzing different tumor models could enhance our understanding of how nanoparticles penetrate tumors.

## 4. Materials and Methods

### 4.1. Reagents

Calcium gluconate solution was provided by Sanchine (Harbin, China). Docetaxel was obtained from Qilu Pharmaceutical Factory (Jinan, China). LysoTracker Red DND-99 and Fluo-4 were obtained from Thermo Fisher (Hillsboro, OR, USA). The apoptosis assay kit was purchased from Multi Sciences (Hangzhou, China). The Cell Counting Kit-8, 1-ethyl-3-[3-(dimethylamino) propyl] carbodiimide hydrochloride (EDC), N-hydroxysuccinimide (NHS), and Indocyanine Green (ICG) were purchased from Sigma (St. Louis, MO, USA). Primary antibodies, including against human albumin (ab207327, 1:500), PV-1 (ab81719, 1:200), Caveolin-1 (ab192869, 1:300), and SPARC (ab225716, 1:200) were purchased from Abcam (Cambridge, UK).

### 4.2. Cell Culture

A liver cancer cell line (MHCC 97-H) was obtained from the China Center for Type Culture Collection (Wuhan, China). The cell line was cultured in Dulbecco’s modified Eagle medium (DMEM) supplemented with 10% fetal bovine serum, 100 IU/mL penicillin, and 100 μg/mL streptomycin. Cells were maintained at 37 °C in a humidified atmosphere with 5% CO_2_ and passaged when confluence reached approximately 80%, typically every 2–3 days.

### 4.3. Biosynthesis of CaO_2_ NPs

Calcium gluconate solution was diluted with sterile water to a concentration of 4 mg/mL, and 1 milliliter of this solution was mixed with 9 mL of human serum and incubated in a shaking incubator at 37 °C for 48 h. The resulting mixture was transferred into ultracentrifuge tubes using a sterile syringe, ensuring the expulsion of all air; the tubes were then sealed using a heat-sealing method. The sealed tubes were symmetrically placed in an ultracentrifuge (Beckman Coulter, Miami, FL, USA) and centrifuged at 35,000 rpm for 2 h. The precipitate was collected and vacuum-dried to obtain the synthesized CaO_2_ NPs.

### 4.4. Preparation of DTX-Loaded CaO_2_ NPs

For drug loading, 1 mg of DTX (1 mg/mL) was added to the CaO_2_ NP solution (1 mg/mL) and the mixture was incubated for 48 h to load DTX onto the CaO_2_ NPs. After centrifugation at 35,000 rpm for 2 h, the precipitate was collected and vacuum-dried to obtain the DTX@CaO_2_ NPs for further use.

### 4.5. Characterization of CaO_2_ NPs

The CaO_2_ NPs were dissolved in sterile water and dispersed via ultrasonication for 5 min; two drops of the resulting suspension were placed on a copper grid and allowed to air dry. Lattice fringes were observed using high-resolution transmission electron microscopy (JEM-2100, JEOL, Tokyo, Japan). The particle size distribution was measured using a nanoparticle size analyzer (Litesizer^TM^500, Graz, Austria), and UV-Vis absorbance spectra were recorded with a UV-Vis spectrophotometer (UH5300, Hitachi, Tokyo, Japan), fluorescence spectra were obtained using a fluorescence spectrophotometer (Spectrofluorometer FS5, Edinburgh, UK), and Fourier transform infrared (FTIR) spectra were collected with a Fourier transform infrared spectrometer (Nicolet iS20, Thermo Fisher, Hillsboro, OR, USA).

### 4.6. Dot Blot Assay

Protein samples were applied onto a PVDF membrane and allowed to dry at room temperature. The membrane was then blocked with 5% non-fat milk for 2 h. After blocking, the membrane was incubated overnight at 4 °C with the primary antibody. Following primary antibody incubation, the membrane was incubated with a horseradish peroxidase (HRP)-conjugated secondary antibody for 2 h at room temperature. Protein levels were visualized using a chemiluminescent substrate.

### 4.7. Live-Cell Imaging

LysoTracker Red DND-99 was diluted to 50 nM in a serum-free medium and incubated with cells at 37 °C for 30 min. Following this, CaO_2_ NPs were added, and the Ca^2+^ probe Fluo-4 was loaded. The release of Ca^2+^ following the endocytosis of the CaO_2_ NPs by MHCC 97-H cells was monitored using a live-cell imaging system (Carl Zeiss, Celldiscoverer 7, Jena, Germany).

### 4.8. Cytotoxicity Assay

Cell proliferation was assessed using the CCK-8 reagent. Cells were seeded at a density of 5000 cells per well in a 96-well plate and incubated for 24 h. Subsequently, 10 μL of CCK-8 solution was added, and the cells were incubated at 37 °C for 1 h. Absorbance was measured at 450 nm.

### 4.9. Flow Cytometry

The cells were resuspended in 500 μL of 1× Binding Buffer, and 5 μL of AnnexinV-APC and 10 μL of PI were added to each sample. After gentle vortexing, the samples were incubated in the dark at room temperature for 5 min. Apoptosis was analyzed using a BD FACS Calibur flow cytometer (BD Biosciences, Franklin Lakes, NJ, USA).

### 4.10. CaO_2_ NPs Crosslink ICG

0.01 g of ICG was dissolved in 250 μL of DMSO, and CaO_2_ NPs (0.03 g) were dissolved in 1 mL of sterile deionized water. The two solutions were mixed and adjusted to a final volume of 5 mL. In parallel, 0.006 g of EDC and 0.003 g of NHS were each dissolved in 1 mL of sterile deionized water. The EDC and NHS solutions were added to the ICG and CaO_2_ NPs mixture, and the final volume was adjusted to 10 mL. The resulting solution was incubated overnight on a rotating mixer. After incubation, the mixture was centrifuged at 1000 rcf for 10 min, and the supernatant was collected as ICG-CaO_2_ NPs.

### 4.11. Investigation of Targeting Ability of CaO_2_ NPs

All animal experiments were approved by the Nanjing Medical University Ethics Committee (IACUC: 2407028). Tumor-bearing BALB/c nude mice (*n* = 3) were intravenously injected with 10 mg/kg of ICG-CaO_2_ NPs. After 72 h, the mice were euthanized, and their heart, liver, spleen, lungs, kidneys, and tumor tissue were collected and placed in clean dishes. The biodistribution of the ICG-CaO_2_ NPs was analyzed using near-infrared (NIR) imaging with the Xenogen IVIS Spectrum system (Caliper, Newton, MA, USA).

### 4.12. Immunofluorescence Staining

Fresh tumor tissues were used to prepare frozen sections (LEICA, Wetzlar, Germany); the tissue sections were incubated overnight at 4 °C with primary antibodies against Albumin, PV-1, Caveolin-1, and SPARC. Subsequently, the sections were incubated with the corresponding secondary antibodies, and the nuclei were counterstained with (DAPI). After drying, the sections were mounted with an anti-fade mounting medium. The interaction between the CaO_2_ NPs and Caveolin-1, PV-1, or SPARC proteins in the tumor tissue was visualized using a laser confocal microscope (Carl Zeiss, Jena, Germany).

### 4.13. In Vivo Antitumor Activity Study

The tumor-bearing nude mice (MHCC 97-H) were randomly assigned to four groups, each consisting of three mice: (1) Ctrl, (2) CaO_2_ NPs, (3) DTX, and (4) DTX@CaO_2_ NPs. When the tumor volume reached approximately 100 mm^3^, each group of mice received intravenous injections on days 0, 4, 8, 12, 16, 20, 24. On day 28, the tumors were excised, and their volumes were measured. Tumor volume (V) was calculated using the following formula: V = (length × width^2^)/2.

### 4.14. Hemolysis Assay

Mouse blood was collected into anticoagulant-treated tubes and centrifuged at 3000 rpm for 15 min. The supernatant was discarded, and the red blood cell pellet was resuspended in an appropriate volume of physiological saline to prepare a red blood cell suspension. The red blood cell suspension was incubated with deionized water, PBS, or varying concentrations of CaO_2_ NPs at 37 °C for 12 h. After incubation, the mixture was centrifuged at 5000 rpm for 15 min, and 100 μL of the supernatant was collected to measure the absorbance at 540 nm. Deonized water served as the negative control and PBS served as the positive control. The hemolysis rate was calculated using the following formula:

Hemolysis (%) = [(OD_samples_ − OD_PBS_)/(OD_deionizedwater_ − OD_PBS_)] × 100%

### 4.15. Biosafety Assessment of CaO_2_ NPs

Six-week-old female BALB/c mice (*n* = 3) were intravenously injected with CaO_2_ NPs on days 0, 7, 14, 21, 28, 35, and 42. Body weight was recorded every 2 weeks. After 6 weeks, the mice were euthanized, and the major organs (heart, liver, spleen, lungs, and kidneys) were harvested and fixed in 10% formalin. Tissue samples were subsequently embedded in paraffin, sectioned, and stained with hematoxylin and eosin for histopathological analysis.

### 4.16. Statistical Analysis

Data are presented as means ± standard deviation (SD). One-way ANOVA with the Tukey post hoc test was used for comparisons among multiple groups (GraphPad Prism 10, Boston, MA, USA). A *p*-value of less than 0.05 was considered statistically significant.

## 5. Conclusions

For the first time, we have identified CaO_2_ NPs as a novel form of calcium ions present in the human body. The biosynthetically derived CaO_2_ NPs exhibit excellent tumor-targeting abilities and favorable biocompatibility, making them a promising platform for drug delivery. This approach holds significant potential to enhance liver cancer therapies in the future.

## Data Availability

Data will be made available on request.
